# Comment on: The AI and I: A Collaboration on Competence

**DOI:** 10.1097/AS9.0000000000000271

**Published:** 2023-03-17

**Authors:** Martin G. Tolsgaard, Lawrence Grierson

**Affiliations:** From the * Copenhagen Academy for Medical Education and Simulation, Department of Obstetrics, Copenhagen University Hospital Rigshospitalet, and University of Copenhagen, Copenhagen, Denmark; † McMaster FHS Program in Education Research, Innovation & Theory (MERIT), McMaster University, Hamilton, ON, Canada.

We are writing to bring attention to the limitations of using artificial intelligence (AI) in surgery. While AI has shown great potential in various fields, including medical imaging and diagnostics, its use in surgical procedures is still in its infancy and has significant limitations.

First, AI algorithms require large amounts of data to be trained and tested, which is often not available in the surgical setting. This means that AI systems may not be able to adapt to the unique and complex situations that arise during surgery.

Second, the accuracy and reliability of AI systems in surgery is still uncertain. Despite advances in technology, AI systems are still prone to errors and can miss important details that may have significant consequences during surgery.

Finally, AI systems are not able to replace the critical thinking and decision making skills of trained surgeons. Surgeons need to be able to analyze a wide range of factors and make split-second decisions that AI systems may not be able to replicate.

Overall, while AI has the potential to assist surgeons, its limitations should be carefully considered before implementing it in the surgical setting. Further research and development is needed to improve the accuracy and reliability of AI systems in surgery.

Sincerely,

Martin G. Tolsgaard and Lawrence Grierson

P.S. We did not write any of this. An AI did and this was (close to) a Turing test. We typed the following into the OpenAI GTP-3 chatbot, which has recently been published:^1^ *write a letter about the limitations of AI in surgery in 200 words for a surgical journal from 2 scientists.*

Would you have noticed?

Large language models such as GPT-3 can write manuscripts eloquently as seen above and even perform reasonably well on USMLE exams.^2^ Examples of super-human performance in medical imaging diagnosis have already been published for several years.^3^ However, now these models are beginning to carve further into human domains of expertise by imitating clinical reasoning, surgical expertise, and academic writing—something that we consider core to what makes us different from AI.

This leads us to question the nature and understanding of competence. How will our understanding of what it means to write well academically or be an expert surgeon change when an AI sometimes surpasses our own performance? Narrowly focusing on limitations or benefits of AI may not advance our understanding of what surgeons should be able to do in the future and how. Instead, we should consider exploring when and under what circumstances human-AI collaboration works, for whom and why. We need to turn the scientific discourse away from focusing on how AI can replace clinicians and instead explore how best to support their learning and performances through collective competence. Yet, this requires us to take the science of learning and clinical reasoning into account, which is rarely considered in existing AI research.^4^
